# Ground Displacement in East Azerbaijan Province, Iran, Revealed by L-band and C-band InSAR Analyses

**DOI:** 10.3390/s20236913

**Published:** 2020-12-03

**Authors:** Sadra Karimzadeh, Masashi Matsuoka

**Affiliations:** 1 Department of Remote Sensing and GIS, University of Tabriz, Tabriz 5166616471, Iran; 2 Institute of Environment, University of Tabriz, Tabriz 5166616471, Iran; 3 Department of Architecture and Building Engineering, Tokyo Institute of Technology, 4259-G3-2 Nagatsuta, Midori-ku, Yokohama 226-8502, Japan; matsuoka.m.ab@m.titech.ac.jp

**Keywords:** L-band PALSAR-2, ground subsidence, synthetic aperture radar, East Azerbaijan Province

## Abstract

Iran, as a semi-arid and arid country, has a water challenge in the recent decades and underground water extraction has been increased because of improper developments in the agricultural sector. Thus, detection and measurement of ground subsidence in major plains is of great importance for hazard mitigation purposes. In this study, we carried out a time series small baseline subset (SBAS) interferometric synthetic aperture radar (InSAR) analysis of 15 L-band PALSAR-2 images acquired from ascending orbits of the ALOS-2 satellite between 2015 and 2020 to investigate long-term ground displacements in East Azerbaijan Province, Iran. We found that two major parts of the study area (Tabriz and Shabestar plains) are subsiding, where the mean and maximum vertical subsidence rates are −10 and −98 mm/year, respectively. The results revealed that the visible subsidence patterns in the study area are associated with either anthropogenic activities (e.g., underground water usage) or presence of compressible soils along the Tabriz–Shabestar and Tabriz–Azarshahr railways. This implies that infrastructure such as railways and roads is vulnerable if progressive ground subsidence takes over the whole area. The SBAS results deduced from L-band PALSAR-2 data were validated with field observations and compared with C-band Sentinel-1 results for the same period. The C-band Sentinel-1 results showed good agreement with the L-band PALSAR-2 dataset, in which the mean and maximum vertical subsidence rates are −13 and −120 mm/year, respectively. For better visualization of the results, the SBAS InSAR velocity map was down-sampled and principal component analysis (PCA) was performed on ~3600 randomly selected time series of the study area, and the results are presented by two principal components (PC1 and PC2).

## 1. Introduction

Since urban growth in recent decades has accelerated on a global scale, migration of the population from rural to urban areas has also increased. This transformation of the population has caused many changes in which the elements of a simple rural life have been gradually replaced by more complex elements that need more resources to meet the demands of a new urban life in an era of complexities. New challenges need to be recognized and modeled before they become problems that can harm other elements or components of environmental systems [1,2,3,4,5]. The first census of Iran in 1956 reported 201 cities, which increased five times by 2011. According to the 1976 census, Tehran was the only city with a population over 500,000, but in the 2011 census, 14 cities, including Tabriz, Mashahd, and Isfahan, had a population of more than 500,000, which means that urbanization and industrialization plans were pursued in recent decades [6].

Ground subsidence is gradually becoming a major problem for governments and people, especially if not properly planned industrial, mining, and agricultural activities are accelerated [7,8,9,10,11,12,13]. It is a long-term phenomenon in most cases and acts like a “dark death” for the Earth. It commonly happens in different parts of Iran. For example, important basins in Tehran, Mashhad, Rafsanjan, Yazd, and Neyshabour have fallen victim to ground subsidence due to a lack of underground water recharge, a lack of supervision, and illegal use of natural resources [14,15,16,17,18].

Optical imagery suffers from the presence of clouds and night/day cycle, but synthetic aperture radar (SAR) imagery provides an opportunity to observe the Earth during the day and at night under all weather conditions. New satellite constellations of SAR missions such as Cosmo-SkyMed, TerraSAR-X, and ALOS-2 can guarantee high-resolution SAR data for different applications. SAR interferometry (InSAR) is a powerful technique that uses the phase and amplitude information of at least two SAR images with the same geometry and characteristics to extract the displacement rate of earthquakes [19,20,21,22,23,24,25,26], ground subsidence [14,15,16,17,18], volcanic activity [27,28,29,30], mining activities [7,31], structures [32,33,34], and landslides [35,36,37,38,39,40,41,42]. The technique enables us to overcome the challenges of conventional methods. For example, ground displacements can be monitored by the Global Navigation Satellite System (GNSS) or leveling, which are point-like conventional methods that require more labor and a considerable budget to maintain a network [43,44]. InSAR has fair spatial coverage without maintenance costs and provides acceptable results for different time spans.

This study presents the spatiotemporal characteristics of intensity, phase, and coherence of PALSAR-2 L-band images (λ ~24 cm) of the ALOS-2 satellite provided by the Japan Aerospace Exploration Agency (JAXA) at different times and focuses on the small baseline subset (SBAS) InSAR technique and principal component analysis (PCA) in NW Iran (East Azerbaijan Province) to monitor the ground subsidence of major basins using 15 PALSAR-2 images.

## 2. Study Area and Datasets

### 2.1. Study Area Description

**Figure 1:**
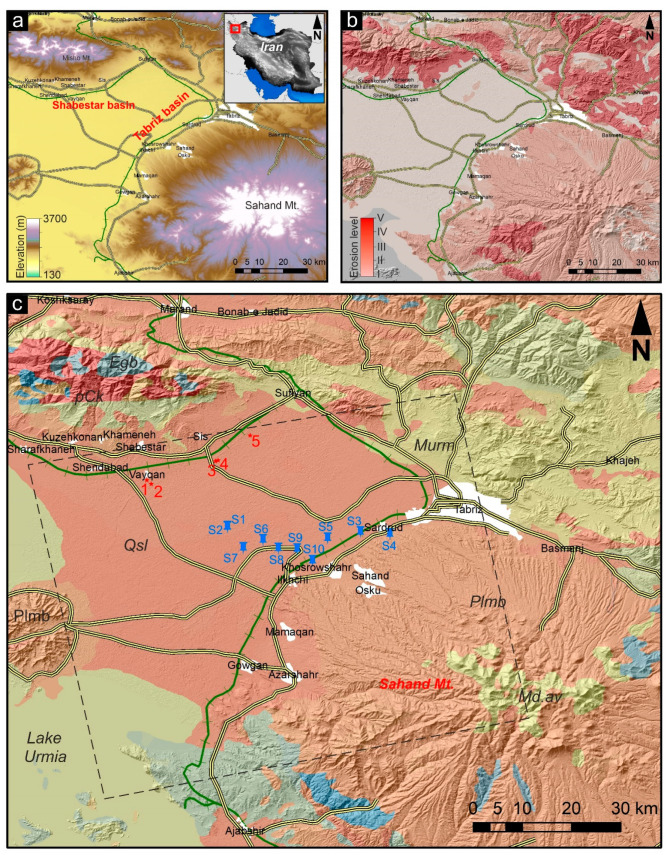
(**a**) Topographic map of study area from Shuttle Radar Topography Mission (SRTM 1 arc-second). (**b**) Erosion map of study area provided by Geological Survey of Iran (GSI) on shaded relief SRTM 1 arc-second. (**c**) Study area with geological units provided by GSI on shaded relief SRTM 1 arc-second. White polygons are cities, red stars and numbers are locations of field observations, blue pushpins are locations of piezometers, dashed square indicates ascending footprint of L-band PALSAR-2 images. Green and thick yellow lines are main railways and roads, respectively. Qsl, Quaternary (unconsolidated sediment); Md.av, dacitic to andecitic subvolcanic rocks; Murm, red to brown marl with gypsiferous marl with sandstone; Plmb, pyroclastic and claystone with vertebrate fauna remains; pCk, dull green-gray salty shales; Plmb, ash-flows; Egb, gabbro rock.

The present study mainly focuses on two major plains in East Azerbaijan Province of northwestern Iran, Shabestar and Tabriz basins, with an area of approximately 1000 km^2^. As shown in Figure 1a, the study area has a harsh topography, and accordingly, there is a concentration of agricultural activities in these flat basins due to their fertility and suitability for different activities. Shabestar and Tabriz basins have been known for intensive agricultural and industrial practices for several decades [45,46,47,48]. The substrate of these basins contains various formations such as gypsiferous marl and Quaternary units [13]. The basins contain abundant unconsolidated Quaternary deposits and a lower erosion level (Figure 1b). As shown in Figure 1b, the level of erosion and soil loss are related to the topography, in which steep positions present a higher erosion level [49]. According to the geological map of the study area provided by the Geological Survey of Iran (Figure 1c), the area of Quaternary deposits inside the PALSAR-2 footprint is higher than in other units. Approximately 2500 km^2^ inside the PALSAR-2 footprint contains unconsolidated Quaternary units, whereas the Quaternary units near Lake Urmia changed to salt flats. The second largest unit in terms of area is around 900 km^2^ for pyroclastic and claystone units over the Sahand volcano. Figure 1c shows that major roads and railways are mainly extended over Quaternary deposits in the Shabestar and Tabriz basins, which generally implies a high risk of damage to infrastructure due to ground subsidence. The Tabriz basin is a hotspot of agricultural and industrial activities in NW Iran, with many legal and illegal pumping wells. Some of the legal piezometric wells are shown in Figure 1c, indicated by blue pushpins.

### 2.2. Dataset Description

Previous studies show that using 17 ENVISAT C-band images (λ ~6 cm), the ground subsidence rate in the Tabriz basin was extracted by the SBAS method for a 7-year period from 2003 to 2010 [45,46], in which the maximum ground subsidence rate was −20 mm/year in the line of sight (LOS) of the satellite. Furthermore, these studies reported that the water level during those 7 years declined up to 1.5 m in some of the piezometric wells, which resulted in three oval-shaped subsidence patterns in the Tabriz basin [45,46]. For Shabestar basin, 14 ENVISAT C-band images from 2003 to 2010 were processed based on the SBAS technique, and the results revealed that the maximum rate of subsidence was about −40 mm/year in the LOS direction [47]. There are several InSAR techniques, depending on the topographic situation, extent of the study area, and number of images, and different InSAR techniques might be selected for crustal displacement analysis [12]. This work used SBAS InSAR time series analysis for L-band data acquired from 5 December 2015 to 4 April 2020. As shown in Table 1, the PALSAR-2 and Sentinel-1 datasets were used. Generally the continuity of L-band (PALSAR-2) data is lower than C-band (Sentinel-1) data, but L-band data has the advantage of strong intensity correlation and long-lasting temporal coherence in order to perform more accurate time series InSAR analysis [50].

**Figure 2:**
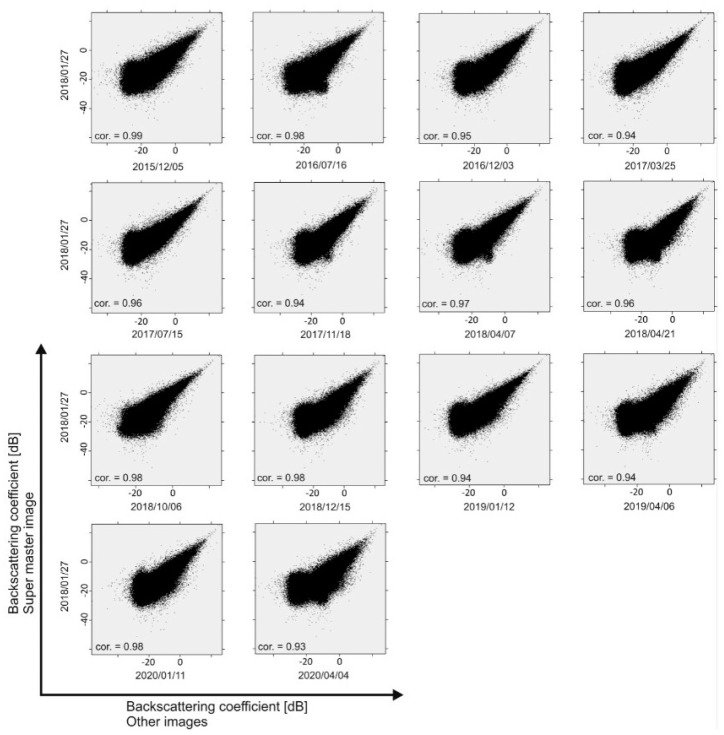
Scatter plots of backscattering coefficients of PALSAR-2 images with respect to super master image.

**Figure 3:**
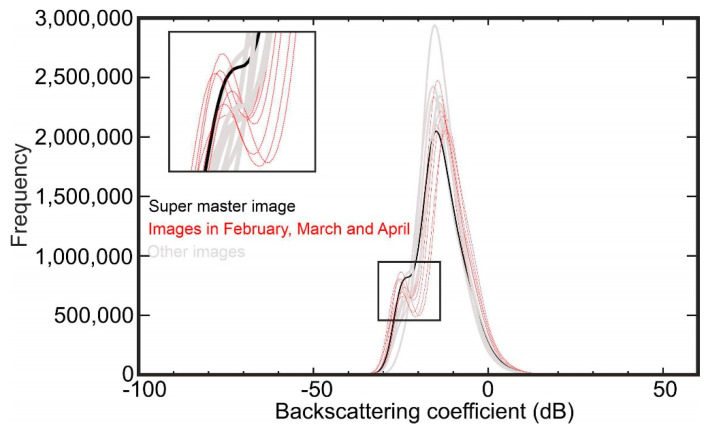
Histogram of PALSAR-2 images used in this study.

Figure 2 shows the correlation of backscattering values (dB) of all PALSAR-2 images with the super master image (27 January 2018). Overall, the correlation of all images with the super master image is high, but images from the same month or season as the super master image have much higher correlation, indicating that the vegetation effects of the backscattering coefficients are probably similar. For example, the correlation between images 6 (super master image) and 13 is 0.98, since their seasonal difference is only 16 days regardless of what year they were acquired. In addition, Figure 3 shows a histogram of the backscattering coefficients of the target area, in which the majority of images show a normal distribution (bell-shaped curve). The shape of the distribution is important in natural sciences to understand the physical situation of the study area [26]. Figure 3 shows that the peak of the bell curve for all PALSAR-2 images is formed between −5 and −15 dB. The normal distribution is generally symmetric near the mean value and is non-zero over the real line. However, some curves in Figure 3 are not exclusively symmetric (red curves). For example, histograms of the images acquired in February, March, and April show sinusoidal behavior in the left shoulder of the bell curve, mainly because of considerable rain. Other images, including the master image, that were acquired in dry seasons or months show relatively symmetric bell curves.

Although the main focus of this work is SBAS analysis of L-band PALSAR-2 data, conventional differential InSAR (DInSAR) analysis was also applied on the C-band Sentinel-1 dataset, for comparison or validation of the L-band SBAS results. The dual polarized horizontal (H) and vertical (V) PALSAR-2 data (HH + HV) were acquired from ascending orbits (right-looking observations) of track 178, while the dual polarized Sentinel-1 data (VV + VH) were acquired from descending orbits (left-looking observations) of track 79. The averaged incidence angle difference (Δθ) of the two datasets for all interferometric pairs did not exceed 0.02°, which implies that the acquisition positions were ideal.

## 3. Methods

The displacement is extracted in two stages: SBAS time series analysis and PCA.

### 3.1. SBAS Analysis

Several SBAS InSAR time series studies based on different X-band and C-band datasets have been done for NW Iran [45,46,47,48,51]. A small baseline selection is based on SBAS interferogram calculation. We assume that 15 PALSAR-2 images make up a set of single-look complex (SLC) images (*N*) over the same area, with the same imaging geometry from the same orbit acquired at ordered times (*t*_1_, *t*_2_, *t*_3_, *…*, *t_n_*). The quality of interferogram correlation between 2 selected images is a product of the time period (*T*), the normal baseline of the two acquisitions (*B*), the Doppler centroid of the 2 images (*D*), and the thermal noise imposed by the sensor [52]. Thus, the conventional form of SAR interferometry is as follows:

(1) $$\rho =\rho_{temporal}\cdot \rho_{spatial}\cdot \rho_{Doppler}\approx (1-f(T_{⊥}-T_{⊥}^{c})).\;(1-f(B_{⊥}-B_{⊥}^{c})).\;(1-f(D_{⊥}-D_{⊥}^{c}))$$

where ρ is the correlation, $T_{⊥}$, $B_{⊥}$, and $D_{⊥}$ are temporal, normal interferometric, and Doppler baseline respectively, and $T_{⊥}^{c}$, $B_{⊥}^{c}$, and $D_{⊥}^{c}$ are critical temporal, critical normal, and Doppler centroid baseline, respectively. If the correlations defined by Equation (1) meet the SBAS criteria, the corresponding SAR pair can be selected for interferogram generation of small baseline analysis. Instead of following Zebker et al. [52], here, for the SBAS analysis, we followed Berardino et al. [53]. For this analysis, the minimum number of pairs is 1 for 2 images and each interferogram consists of 2 images. Thus, the amount of phase value can be estimated considering *N* as the odd number of images and *M* as the number of interferograms:

(2) $$\frac{N+1}{2}\leq M\leq N\left(\frac{N+1}{2}\right).$$

Since the pixel values of SAR images are in radar coordinates, not geodetic/geographic coordinates, the pixel value of unwrapped interferogram j at times $t_{B}$ and $t_{A}$ in the azimuth and range direction will be as follows [53]:

(3) $$\varphi_{j}\left(x,\;r\right)=\varphi \left(t_{B},\;x,\;r\right)-\varphi \left(t_{A},\;x,\;r\right)\approx \frac{4\pi }{\lambda }\left[d^{LP}\left(t_{B},\;x,\;r\right)-d^{LP}\left(t_{A},\;x,\;r\right)\right]+\Delta \varphi_{j}^{atm}\left(t_{B},\;t_{A},\;x,\;r\right)+\Delta \varphi_{j}^{topo}\left(x,\;r\right).$$

where j is an integer number from 1 and M, $\varphi \left(t_{B},\;x,\;r\right)$ and $\varphi \left(t_{A},\;x,\;r\right)$ are phases of 2 SAR images looked at multiple times at times $t_{B}\;$ and $t_{A}$ in the range and azimuth direction, $d^{LP}\left(t_{B},\;x,\;r\right)$ and $d^{LP}\left(t_{A},\;x,\;r\right)$ are displacement values of low-pass component for a time span from $t_{A}\;$ to $t_{B}$ in the LOS of the satellite, $\Delta \varphi_{j}^{atm}\left(t_{B},\;t_{A},\;x,\;r\right)$ is the associated phase component of atmosphere from $t_{A}\;$ to $t_{B}$, the wavelength of the satellite is represented by λ, and $\Delta \varphi_{j}^{topo}\left(x,\;r\right)$ is the phase component of topography, which is defined as follows:

(4) $$\Delta \varphi_{j}^{topo}\left(x,\;r\right)\approx \frac{4\pi }{\lambda }\frac{B_{⊥j}\Delta z\left(x,\;r\right)}{rsin\theta },$$

where $B_{⊥j}$ is the perpendicular (normal) baseline of the two SAR acquisitions, θ is the incidence angle of the SAR images, which is approximately 28° for PALSAR-2 images used in this study, and Δz(x, r) is a topographic artifact that can be reduced by a digital elevation model (DEM).

Here, thermal noise is assumed to be negligible, and according to Equation (3), the topography and atmosphere are non-displacement phase components that must be reduced or removed. Thus, the number of processed pairs and their temporal and spatial baselines should be selected under predefined criteria. Since the L-band PALSAR-2 images can keep high phase correlation over time, we assume that all SAR images are eligible for SBAS analysis, despite the large gaps between image acquisitions. So, the maximum temporal gap for a potential SBAS pair is assumed to be 365 days, and the spatial (normal) baseline eligible for an SBAS pair is up to 45% of the critical baseline shown in Equation (1). Although more SAR images are preferred for SBAS analysis, 15 images is marginally enough [48].

**Figure 4:**
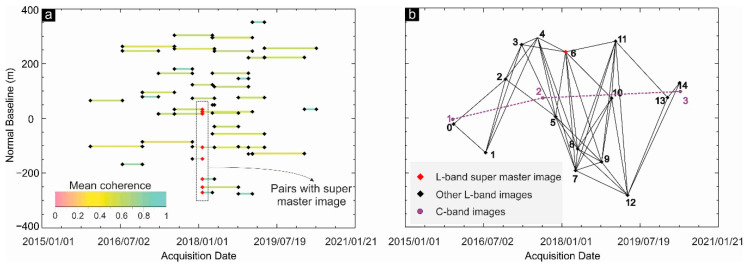
(**a**) Arrangement of interferometric synthetic aperture radar (InSAR) pairs and corresponding mean coherence values for each pair. (**b**) InSAR links of PALSAR-2 (black diamonds) and Sentinel-1 (purple circles). Super master image for small baseline subset (SBAS) analysis of PALSAR-2 data indicated by red diamond.

Figure 4 shows that the criteria applied for SBAS network creation have enough integrity to minimize the errors, as the pairs are continuously connected to each other. The algorithm is adopted to retrieve the highest coherence values, on the one hand, and keep the integrity of the SBAS network for the pairs with coherence higher than 0.8, on the other hand. Therefore, interferograms with a coherence value lower than 0.8 are withdrawn. The minimum and maximum normal baselines are 24 and 352 m, respectively. The selected pairs must be co-registered to one of the images. Image co-registration is a process where 2 or more SAR images have the same geometric nature and corresponding pixels should represent the same objects that might be integrated. This is an essential step for SBAS time series analysis to make sure that pixel values for a certain object are correctly resampled and able to create the same voxel of the objects. We co-register the images to a super master image that is located in a fair position (both temporally and spatially) to the other images.

In Figure 4a, the super master image is indicated by a small red diamond in the middle of the network that is used to generate 8 interferograms. Moreover, it can satisfy the major temporal and spatial limitations of time series analysis as it is located in fair position with respect to the other images (Figure 4b). Overall, 86 pairs were analyzed, and their average coherence values are shown in Figure 4a. Generally, short pairs have higher mean coherence values and long pairs have lower mean coherence values: most of the pairs have an average coherence value between 0.5 and 0.8. The 1 arc-second Shuttle Radar Topography Mission (SRTM) DEM (30 m resolution) along with 50 ground control points (GCPs) were used for removal of topographic and atmospheric phases. The mean coherence map of all pairs, the topography map, and interferograms are criteria used for the selection of GCPs in highly coherent areas with smooth topography, far from the subsiding areas.

The method described above is efficient when geodetic measurements are limited or not available. Once the displacement map in LOS is generated and reprojected in a geographic/geodetic coordinate system, in order to compare the results of PALSAR-2 with other datasets, such as Sentinel-1, the LOS values can be transformed into vertical values. The transformation of coordinates is done by assuming a stable (motionless) area in the results, selected based on high coherence values, away from the subsiding areas. For a three-dimensional displacement map, both ascending and descending datasets of PALSAR-2 are necessary. Since our PALSAR-2 dataset was limited only for the ascending dataset, the retrieval of vertical values could be done under several assumptions. The SAR imaging system is not highly sensitive to north–south movements due to its pole-to-pole movements. We assume that displacements in the N–S and E–W directions are negligible and only the vertical displacement can be inferred, as follows:

(5) $$V_{v}=\frac{V_{LOS}+\;V_{EW}sin\theta cos\alpha }{cos\theta }$$

where $V_{v}$ is the vertical displacement rate, $V_{LOS}$ is the LOS deformation rate, $V_{EW}$ is the east‒west displacement rate, α is the azimuthal angle of the LOS, and θ is the incidence angle of the satellite.

### 3.2. Principal Component Analysis (PCA)

The results obtained from the SBAS need to be interpretable, since thousands of pixel values have displacement values for a certain time span. PCA is a useful method to analyze time series data in the form of observations (M) and variables (N). It has advantages in allowing us to quickly visualize the results, and to analyze the correlation between variables and observations on a simple map that has a low dimension and an optimal view for a variability criterion. Here, the vertical displacement deduced from SBAS are on matrix or a table, in which the observations are displacements (mm) of different locations and the variables are different acquisition dates. The observations and dates are kept in individual rows and columns of the matrix, respectively. The general form of the PCA in this study can be defined as follows:

(6) $$X=TP^{\prime }+r$$

where X is the PCA matrix that contains the time series, T is a matrix of scores and P is a matrix containing the loading factors (this matrix is transposed), and r is the residual or unexplained portion of the results. It must be noted that PCA originates from a projection method. If the data are too complex, the results may lead to misinterpretation, because PCA does not allow testing of the hypotheses. One solution could be to select different observations or variables from the SBAS results and run the model each time, as long as such manipulations (adding or removing observations/variables) are justified in the interpretation. Here, since the size of the preliminary SBAS matrix was too large, we randomly selected 5000 SBAS time series candidates for the PCA matrix (X). T and P were estimated from the least squares method, and each PC had one score and loading factor.

## 4. Results

### 4.1. Coherence Changes

**Figure 5:**
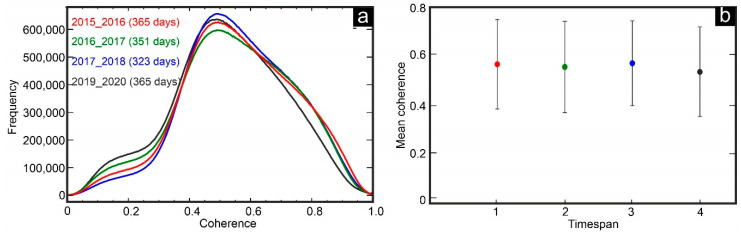
(**a**) Statistical results of coherence changes for SAR pairs with nearly one year time span, and (**b**) mean coherence values of four time spans.

**Figure 6:**
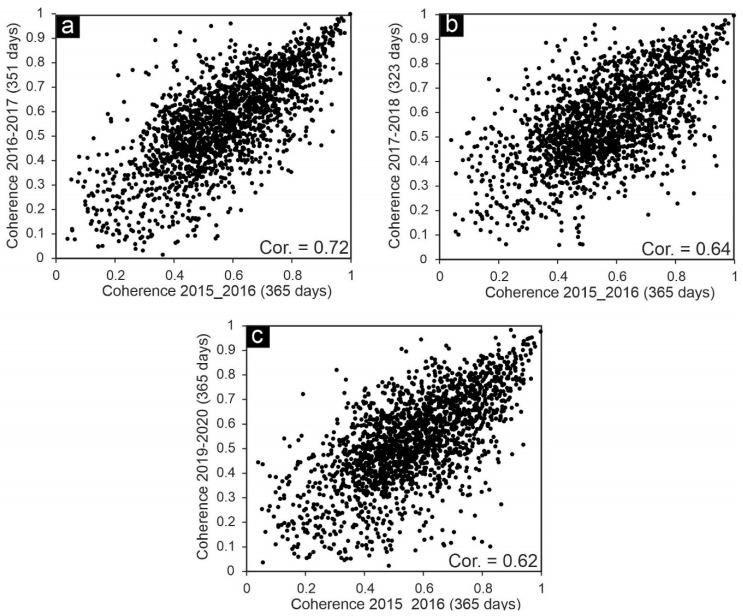
Scatter plots and correlation coefficients of (**a**) time span 1 (2015–2016) against time span 2 (2016–2017), (**b**) time span 1 (2015–2016) against time span 3 (2017–2018), and (**c**) time span one (2015–2016) against time span four (2019–2020).

Figure 5a shows spatiotemporal changes of the coherence histograms of four time periods, in which the corresponding time span is nearly one year: time span 1 (365 days), time span 2 (351 days), time span 3 (323 days), and time span 4 (365 days). The mean coherence values of time spans 1, 2, 3, and 4 are 0.559, 0.548, 0.563, and 0.528 and the standard deviations are not small regarding the calculated mean values. The standard deviations are 0.182, 0.186, 0.173, and 0.183, respectively (Figure 5b). Although the general behavior of the coherence maps is similar, the peak position of the third period (18 November 2017 to 6 October 2018) is higher than the others, probably because of its shorter temporal gap (Figure 5a). The mean value of time spans shows that as the temporal gap between two images increases, the mean coherence value decreases. Figure 6 shows a cross comparison of time span 1 with time spans 2, 3, and 4.

### 4.2. InSAR Displacements and Field Observations

**Figure 7:**
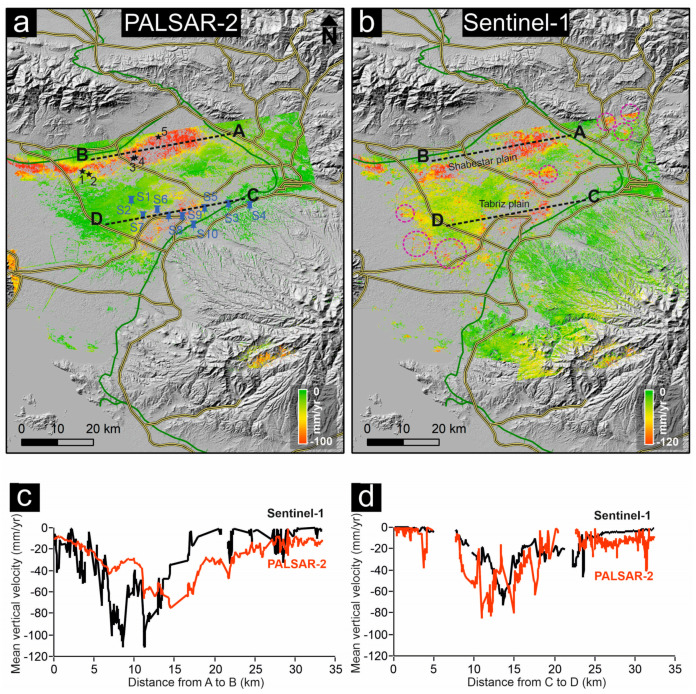
Mean vertical velocity map of study area from (**a**) PALSAR-2 L-band and (**b**) Sentinel-1 C-band during 2015–2020, (**c**) displacement profile from A to B, and (**d**) displacement profile from C to D. Black stars show locations where field observations were carried out (see Figure 9). Blue pushpins in (**a**) show locations of wells where regular groundwater measurements are available. Purple circles in (**b**) are displacement, atmospheric, or topographic phases that are not observed in (**a**). Thick green and yellow lines indicate railway and first-degree networks, respectively.

**Figure 8:**
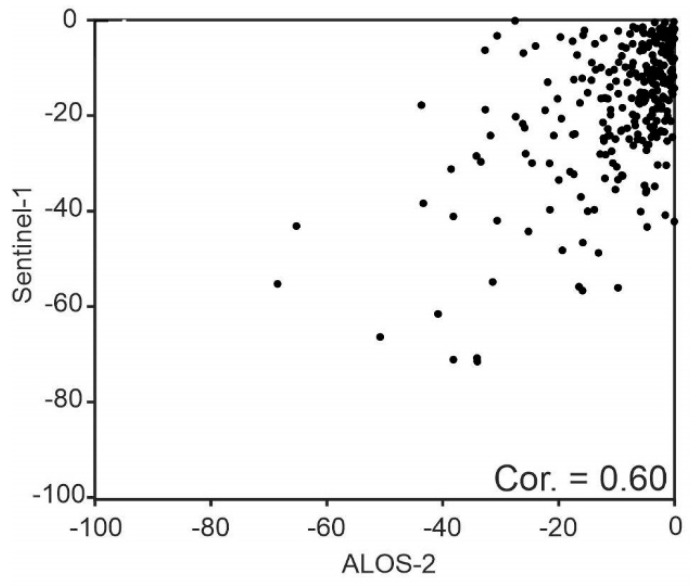
Scatter plot of PALSAR–ALOS-2 versus Sentinel-1 velocities.

**Figure A1:**
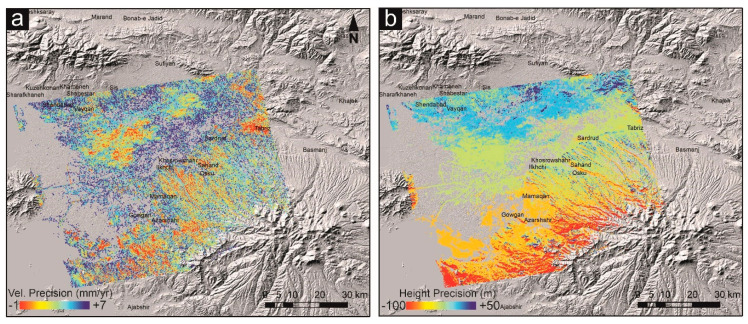
(**a**) Velocity precision of SBAS analysis and (**b**) height precision of SBAS analysis.

Figure 7a,b shows the mean vertical velocity of SBAS results extracted from LOS rates and Equation (5) for PALSAR-2 L-band ascending images and conventional velocity map of C-band (Sentinel-1) descending images, respectively. There are two main displacement features in results of both L-band and C-band datasets with an NE–SW trend in Shabestar and Tabriz basins. Although velocity precision and height estimations during SBAS analysis show that some uncertainties may still remain in the results (Appendix A, Figure A1), the associated motions in the two datasets suggest a dominant long-term downward movement peaking at up to −100 and −120 mm/year, respectively. Figure 7c, d shows the ground subsidence rate along profiles A–B and C–D (shown in Figure 7a,b) from east to west. The A–B section for Sentinel-1 and PALSAR-2 shows good agreement, but at some locations, there are differences up to 4 cm. The subsidence rate in the first 12 km of profile A–B is higher in the Sentinel-1 results. On the contrary, the subsidence rate in the rest of the profile is higher in the PALSAR-2 results. In profile C–D, the results of PALSAR-2 and Sentinel-1 also show a similar pattern. Since the time spans of PALSAR-2 and Sentinel-1 datasets are very close, the reason for the cm-scale difference between their results in section A–B might be related, first, to the temporal decorrelation of the Sentinel-1 dataset and the high level of uncertainty in conventional InSAR analysis. Second, the wavelength of PALSAR-2 images is longer than that of Sentinel-1 images, and the sensitivity of the two datasets is different in detecting ground displacements. The correlation between PALSAR-2 and Sentinel-1 results along the profiles A–B and C–D, are 0.81 and 0.85, respectively. We also compared the SBAS InSAR map of PALSAR-2 data and the InSAR map of Sentinel-1 data for the whole study area. Figure 7 shows a cross comparison of the results. The correlation of the two maps is 0.6, and the mean vertical rate of ground subsidence and standard deviation for PALSAR-2 results are −10 and 9 mm/year, respectively (Figure 8). For Sentinel-1, the mean vertical rate and standard deviation are −13 and 12 mm/year, respectively.

**Figure 9:**
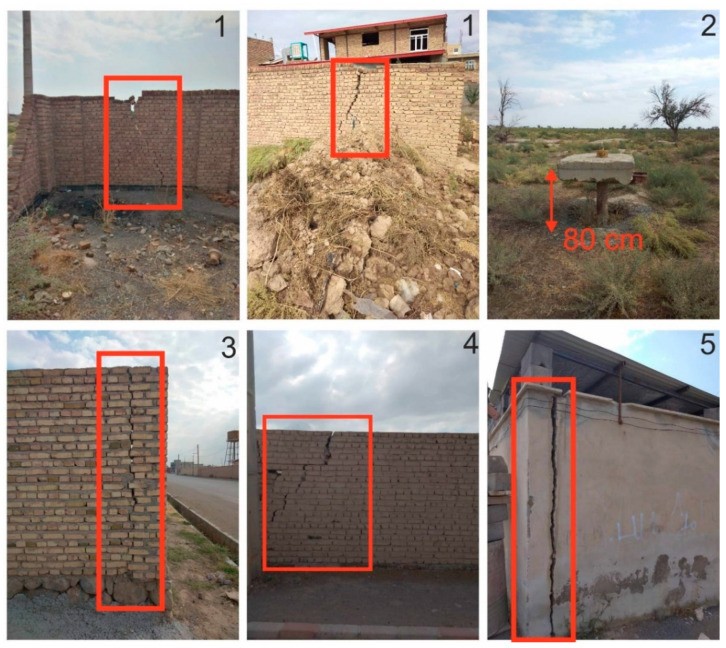
Subsidence evidence at different locations in Shabestar basin. Locations of images are shown in Figure 1c and Figure 7a.

Geodetic measurements such as Global Positioning System (GPS) and precise leveling in the study area are lacking, and we only gathered time series measurements of piezometric wells in the Tabriz plain. In addition, we gathered field evidence in the Shabestar plain. As shown in Figure 9, many buildings in the Shabestar basin and adjacent areas have been damaged as a result of progressive ground subsidence. Site 2 in Figure 9 (see its location in Figure 1c and Figure 7a), an old piezometric well that was established approximately four decades ago, subsided at least 80 cm. Although the subsidence evidence around the old piezometric well is clear, it is unknown when the subsidence rate was accelerated. Some infrastructure in Shabestar city and two small towns, Shendabad and Vayqan (see their location in Figure 1), are also at immediate risk of ground subsidence.

We drew a buffer of 5 km around the railways and major roads, and their proximity to the subsidence rates is presented in Table 2. The Shabestar basin is an unconfined aquifer with approximately 800 wells, 6 springs, and 161 qanats (traditional water extraction system in Iran), from which the extracted water in 2014 was estimated to be around 88 × 10^6^ m^3^ [13]. The number of deep wells not only in the study area but also in other large basins of Iran, such as Tehran basin, has increased. The latest public report shows that the number of legal/illegal wells in Tehran increased more than eight times over 44 years (1968 to 2012) [54]. In Tabriz plain, the situation is the same, but the exact volume of extracted water is unknown due to many illegal deep-water pumping wells.

**Figure 10:**
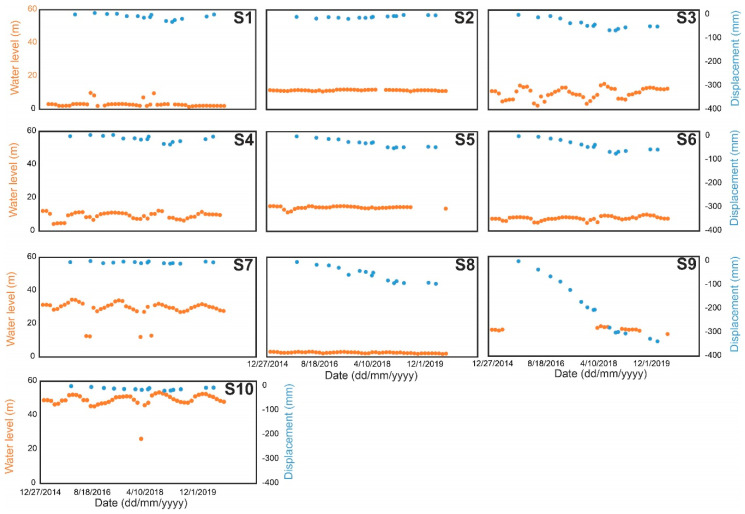
Vertical water changes versus vertical InSAR at 10 locations. Locations of wells are shown in Figure 1c and Figure 7a.

In Tabriz plain, we obtained groundwater information of 10 piezometric wells as gathered by the regional water organization (RWO). Regular monthly measurements of the water level in Tabriz basin are available since 2001. We only plotted groundwater time series from 2015 to 2019. The water fluctuation and ground subsidence (SBAS results) at the same level along with the linear regression are shown in Figure 10. The level and behavior of piezometric wells are different. Wells S1, S2, and S2 have a very low water level ranging from 0 to 4 m, probably because they are not deep enough to conduct accurate measurements, while some wells, such as S7 and S10, have a higher water level ranging from 20 to 55 m. In some wells, such as S3, S7, and S10, seasonal fluctuation is evident, while in wells such as S5, the water level was stable or gently rose from 2015 to 2020. One reason for the gentle water rise in the subsiding area could be related to the synthetic recharge of wells by sewage water of large infrastructure such as the Tabriz petrochemical factory or power plant.

A comparison between water level and InSAR displacement in Figure 10 shows that ground subsidence matches with water level decline in some of locations, such as S8 and S9. Unfortunately, water measurements at S9 were not completed and there are some gaps without regular measurement. In other locations far from the subsiding area, such as S4 and S10, ground water fluctuation and InSAR displacement are in good agreement, which implies that the ground water level and InSAR displacement have a direct relationship. Nevertheless, in locations such as S3, ground displacement does not follow ground water fluctuations. In Tehran basin, the same issue happened in some piezometric wells [54].

**Figure 11:**
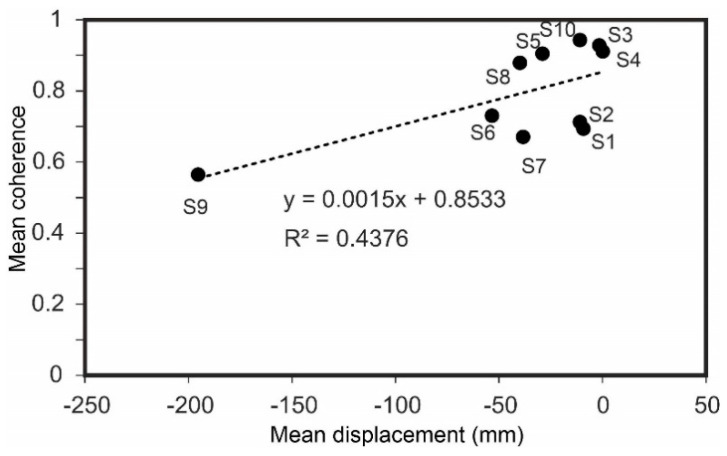
Scatter plot of SBAS mean displacement and mean coherence values at locations of 10 piezometric wells.

At the heart of the subsiding area in Tabriz basin where S9 is located, the mean displacement value is −195 mm, which is the highest subsidence value among the listed wells. In contrast, the corresponding mean coherence value of S9 is about 0.56, which is lower than other known pumping stations. Figure 11 shows that an increased mean coherence value can result in a slower displacement rate. When the mean coherence value is high, it could be an indicator of slower changes in agricultural activities and vegetation growth of the study area. Comparing the spatial distance of the wells reveals that closer wells have similar mean coherence values, which implies that soil moisture and geomorphological characteristics of the wells may be related to mean coherence values over the time span. In Figure 11, S1 and S2, at the upper margins of the subsidence area, are spatially close to each other, and not surprisingly, their mean coherence values are 0.69 and 0.71, respectively.

### 4.3. PCA Results

**Figure 12:**
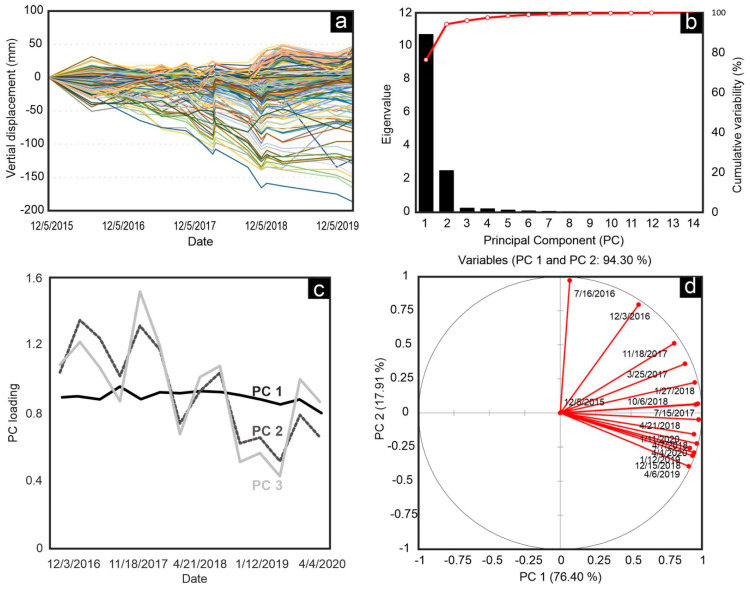
(**a**) SBAS time series of randomly selected points for PALSAR-2 dataset (only 250 points are shown), (**b**) scree plot of principal components (PCs), (**c**) three major components of principal component analysis (PCA), which explain about 96% of the variance, and (**d**) circle plot of variables for PC1 and PC2 loadings.

Several studies proposed that because of the high dimensionality of InSAR time series, it is difficult to study separate crustal movements that may occur simultaneously. For example, seasonal movements and long-term movements occur in the study area, as shown in Figure 12a. However, a conclusive estimation of different trends needs further analysis, such as PCA. PCA may fail for large SBAS results. Hence, we resampled only 10,000 points randomly in different parts of the study area. The results show that the first three principal components (PCs) explain a major part of the SBAS results: PC1 (~76%), PC2 (~18%), and PC3 (~2%) together explain about 96% of the entire variance (Figure 12b). However, some information might be hidden in other PCs.

Each PC corresponds to a specific score value that describes the variation shape. Figure 12c shows PC1, PC2, and PC3 loading values from 2015 to 2020. We can consider PC1 as a long-term component of ground subsidence, which is the largest portion of the variance, while PC2 and PC3 are seasonal changes with several troughs and peaks. The behavior of PC2 and PC3 is complex despite their similar pattern, indicating that seasonal change may have occurred differently between 2015 and 2020. Figure 12d shows a correlation circle of the results, which is a projection of the SBAS variables in the PC space. If two variables are far from the center of the circle and close to each other, then it can be interpreted that they are positively correlated (near +1), and if two variables are perpendicular to each other, they are not correlated (near 0). If two variables are located in opposite directions of the center, they are negatively correlated (near −1). Comparing PC1 and PC2 in Figure 12d, the positive correlation of the results between 2018 and 2020 is higher, probably because the number of obtained images in this period is considerably higher than in 2015–2017.

## 5. Discussion

The correlation coefficient of the coherence between time spans 1 and 2 is 0.72, between time spans 1 and 3 is 0.64, and between time spans 1 and 4 is 0.62 (Figure 6). The high values for time spans 1 and 2 and low values for time spans 1 and 4 are not surprising, because as the temporal gap increases, the correlation coefficient decreases, mainly because of urban or vegetation growth, land use changes, etc. Although the PALSAR-2 with longer wavelengths can preserve the phase correlation in larger timespans, the standard deviation of the above-mentioned time spans is not small. The results of L-band (SBAS) and C-band (DInSAR) both identified two subsidence patterns in Shabestar and Tabriz basins. The mean subsidence rate of the DInSAR method is higher than that of the SBAS method. The velocities deduced from the C-band dataset show that there are higher subsidence rates in central, eastern, and western parts of the study area (dashed circles in Figure 7b), while these parts are almost stable in L-band results (Figure 7a). The strong displacement (overestimation) in these parts is related to the ambiguous nature of the phase [55] in pure displacement, atmospheric component, and remaining topographic phase, etc. For this reason, establishment of GPS stations in different parts of the study area for continuous measurement is essential for accuracy evaluation of DInSAR and SBAS results in future studies.

As stated before, the PCA methodology helped us to find the trend of long-term and seasonal displacements based on the average velocity and the explained variance. PC1 explains ~70% of the variance (Figure 12b) and the corresponding PC loading in Figure 12c gives insight into the correlation between the uniform trend of subsidence in the study area. PC1 is well-explained by PC loading and the land subsidence is evident. But explanation of seasonal behavior is difficult, because PC2 and PC3 are similar. Further statistical tests may help to solve the complexity of the PCs, especially when the results are similar.

## 6. Conclusions

Tabriz and Shabestar basins have experienced ground subsidence for several years, mainly due to heavy water extraction for industrial and agricultural activities, as reported in several previous studies [13,42,43,44]. All of the previous studies presented results of ground subsidence for the years before 2010 using C-band datasets such as ENVISAT tracks 49 and 92. As a fresh step in this study, we presented the ground subsidence rate using the L-band PALSAR-2 dataset. The SBAS results demonstrated that roads and railways are at immediate risk of ground subsidence, in which the maximum vertical subsidence rate for the roads and railway inside the ALOS-2 footprint is −81 and −56 mm/year, respectively. As shown in PCA results, the existence of long-term subsidence (i.e., PC1) in the study area could cause serious damage to the infrastructure. The SBAS ground subsidence rates were compared with the Sentinel-1 ground subsidence map. The displacement rates of both maps for the settlement zones showed a similar pattern and relatively close displacement rates. We concluded that the main urban area in Tabriz is stable according to Figure 7a,b. However, the displacement rates, piezometric measurements, and field observations suggest progressive land subsidence during 2015–2020. We also conclude that in the absence of geodetic measurements, the combination of SBAS and PCA can provide us with an opportunity to extract seasonal and long-term motions using PC loadings and scores. However, with regard to the similarity of PC2 and PC3, a question remains about whether the secular motions and noises are separated appropriately or not. Independent component analysis (ICA) could be conducted in the future to quantify the behavior of PC2 and PC3. This study mainly compared the SBAS vertical rate for 10 locations of piezometric data without in-depth quantitative analysis. Further geological and geotechnical data from boreholes in different locations could reveal more aspects of ground subsidence in the future.

## Figures and Tables

**Table 1:** Synthetic aperture radar (SAR) images and detailed characteristics of L-band PALSAR-2 and C-band Sentinel-1 datasets used in this study. * indicates super master image; D and A indicate descending and ascending orbits, respectively.

Label (#)	Date (YYYY/MM/DD)	Polarization	Incidence Angle (°)	Band	Orbit	Track
0	2015/12/05	HH/HV	28	L-band	A	178
1	2016/07/16	HH/HV	28	L-band	A	178
2	2016//12/03	HH/HV	28	L-band	A	178
3	2016/03/25	HH/HV	28	L-band	A	178
4	2017/07/15	HH/HV	28	L-band	A	178
5	2017/11/18	HH/HV	28	L-band	A	178
6 *	2018/01/27	HH/HV	28	L-band	A	178
7	2018/04/07	HH/HV	28	L-band	A	178
8	2018/04/21	HH/HV	28	L-band	A	178
9	2018/10/06	HH/HV	28	L-band	A	178
10	2018/12/15	HH/HV	28	L-band	A	178
11	2019/01/12	HH/HV	28	L-band	A	178
12	2019/04/06	HH/HV	28	L-band	A	178
13	2020/01/11	HH/HV	28	L-band	A	178
14	2020/04/04	HH/HV	28	L-band	A	178
1	2015/12/05	VV/VH	39	C-band	D	79
2	2017/11/12	VV/VH	39	C-band	D	79
3	2020/04/06	VV/VH	39	C-band	D	79

**Table 2:** Detailed information of affected length of railways and roads in PALSAR-2 and Sentinel-1 displacement maps.

Subsidence Map	Total Affected Major Road (km)	Total Affected Railway (km)	Mean Road Subsidence Rate (mm/Year)	Maximum Road Subsidence Rate (mm/Year)	Mean Railway Subsidence Rate (mm/Year)	Maximum Railway Subsidence Rate (mm/Year)
PALSAR-2 SBAS	383	144	−8	−81	−9	−56
Sentinel-1	383	144	−10	−77	−12	−63
